# Study on the Mechanism of the Reversible Color Change of Polyacrylic Acid Modified Gold Nanoparticles Responding to pH

**DOI:** 10.3390/ma14133679

**Published:** 2021-07-01

**Authors:** Runmei Li, Caixia Zhang, Chen Wang, Yongjuan Cheng, Daodao Hu

**Affiliations:** Engineering Research Center of Historical and Cultural Heritage Protection, Ministry of Education, School of Materials Science and Engineering, Shaanxi Normal University, Xi’an 710119, China; lrm@snnu.edu.cn (R.L.); zxc@snnu.edu.cn (C.Z.); ccw1128@snnu.edu.cn (C.W.); CYJ04240045@163.com (Y.C.)

**Keywords:** immobilized polyacrylic acid, gold nanoparticles, conformational change, intermolecular hydrogen-bonding, reversible aggregation

## Abstract

In view of various explanations regarding the pH response of the nanocomposite of gold nanoparticles (AuNPs) modified with polyacrylic acid (PAA) molecules in reported literature, in this work, AuNPs with a size of 20 nm saturatedly loaded with PAA molecules (AuNPs-PAAs) were used to investigate the following aspects of this issue. We investigated the effects of pH on the stability of AuNPs-PAAs in the presence of salt, CTAB, poly (sodium styrenesulfonate) (PSS), ethanol, and free PAA, respectively. Common techniques were undertaken to evaluate the stability, including UV-Vis spectroscopy, Zeta potential analysis, and TEM. The results show that AuNPs-PAAs could respond to pH variations, having a reversible aggregation-to-disaggregation, accompanying their Zeta potential change. The proposed corresponding mechanism was that this reversible change was attributes to the net charge variation of AuNPs-PAAs induced by a reversible protonation-to-deprotonation of PAA rather than the conformational change. It was found that salt, CTAB, PSS, and free PAA could strengthen the dispersity of AuNPs-PAAs, even though their absolute Zeta potential values were decreased to small values or dropped to nearly zero. This abnormal phenomenon was explained by solvation. It was also found that AuNPs-PAAs have an opposite pH response in aqueous and ethanol solutions, justifying the solvation effect. All these results revealed the conformational stability of PAAs immobilized on AuNPs. The methods and the findings of this investigation give some new insights to understand the pH-response of AuNPs-PAAs composites and the design of AuNPs-PAAs-based functional sensors.

## 1. Introduction

Gold nanoparticles (AuNPs) have attracted a great deal of attention due to their unique physical and chemical properties. They are easily modified using functional molecules. Multifarious stimuli-responsive polymers have been widely used to modify AuNPs. Polyacrylic acid (PAA) is a typical stimuli-responsive polymer that can respond to external pH changes. The carboxyl groups on the PAA chain protonate when the pH in the medium is lower than its pKa, resulting in decreased electrostatic interactions and increased intramolecular hydrogen bonding [1]. As a result, it undergoes step changes in chain conformation, passing from open, fully solvated coils to desolvated globular conformations (but not fully collapsed) [2]. Naturally, PAAs coated on AuNPs are expected to show similar pH-dependent conformational changes to free PAAs, and thus induce a reversible change in the aggregation behavior of AuNPs-PAAs composites.

Although a large amount of research focuses on the pH response of AuNPs-PAAs, there are different opinions on their aggregation behavior as well as the corresponding mechanisms. Based on the results of Zeta potential and DLS determination, Jans et al. [3]. suggested that PAA molecules immobilized on the surface of AuNPs have a conformational change with pH. They believed that PAA chains anchored on the surface of AuNPs were protonated in the range of pH 2–4 and adopted a coiled state. Similarly, Brubaker et al. [4] pointed out that PAA molecules on the surface of AuNPs have a stretched conformation at a higher pH due to hydrogen bonding between the partially protonated PAAs and water molecules, while PAA molecules tend to adopt the collapsed conformation due to reduced hydrogen bonding at a lower pH. On the contrary, Vaknin et al. [5] believed that there was no interaction among AuNP-PAAs at pH ≤ 3 in the bulk phase, implying that there was no hydrophobically conformational change of PAAs on AuNPs. Kitchens et al. [6] attributed the larger hydraulic radius of AuNPs-PAAs at a lower pH to the aggregation of AuNPs-PAAs due to the decreased charge induced by the protonated PAAs, while attributing the smaller size of AuNPs-PAAs at a higher pH to an increased electrostatic interaction induced by deprotonated PAAs. Obviously, they attributed the aggregation of AuNPs-PAAs at a lower pH to lower static interactions rather than the hydrophobic interactions caused by the conformational change of PAAs.

In addition to this, there are also some inconsistent reports about the stability of AuNPs-PAAs in the presence of salts. Jans et al. [3] believed that the combination of Na^+^ ions with water molecules bound to PAAs chains reduced the thickness of the PAAs corona on AuNPs-PAAs, resulting in the aggregation of AuNPs-PAAs in presence of 0.15 mM NaCl. However, the experimental results obtained by Kitchens et al. [6] indicated that AuNPs-PAAs maintained a stable state, even in 0.2 M of borax–hydrochloric acid buffer solution. They ascribed this special stability to the steric hindrance and electrostatic repulsion provided by PAAs immobilized on the negatively charged AuNPs.

The following questions could be drawn from the above discussions. Should the change in aggregation behavior of AuNPs-PAAs with pH be attributed to a static interaction or a hydrophobic interaction? Is the presence of salt beneficial or not to the stability of AuNPs-PAAs? As a matter of fact, the conformational change of PAAs responding to pH depends on various factors, such as the molar mass of PAAs, ionic strength in the medium, and solvents [2]. The study revealed that shorter PAA chains (chain length N < 23) tended to be locally stiffer compared to larger ones (chain length N > 46) regardless of the degree of ionization. Monovalent ions, including Li^+^, Na^+^ and K^+^, could promote the formation of a compact structure of PAAs [7]. However, it was found that salt plays the dual role of screening electrostatic interactions and regulating the polymer charge [8]. On the one hand, the increase in the ionic strength results in a decrease in the strength and range of the electrostatic interactions, but, on the other hand, it results in a larger charge of the polymer molecules because of the screening effect of the salt. Additionally, there are some special effects for immobilized PAA molecules. For example, both immobilized and PAAs with a charged density affect the mobility of water molecules and counterions in PAAs brush, which, in turn, affects the mobility of PAAs [9]. Recently, Srivastava et al. explicitly stated that solvents also affect the electrostatic interaction of polyelectrolytes [10]. In summary, there are multiple factors affecting the pH-dependent conformational change of PAAs and the aggregation behavior of AuNPs-PAAs, and these reasons lead to complications in revealing the mechanism of AuNPs-PAAs aggregation caused by pH.

Here, the changes in aggregation behaviors of AuNPs-PAAs in various special media were determined in situ to reveal the possible relation between these changes and the electrostatic interactions or conformation changes. The special media include a negatively charged polymer (poly (sodium styrenesulfonate) (PSS)), a positively charged surfactant (cetyltrimethyl ammonium bromide (CTAB)), a weak polar solvent (ethanol), a salt (NaCl), and a pH-sensitive polymer (PAA). The findings indicate that there is a strong interaction among PAAs immobilized on AuNPs, which strongly inhibits the conformational change of PAA. The aggregation behavior of AuNP-PAAs is mainly related to electrostatic interactions rather than the hydrophobic interactions between PAAs with collapsed conformation. The protocols and the findings of this investigation give some new insights to understand the pH response of AuNPs-PAAs and for the design AuNPs-PAAs-based functional sensors.

## 2. Experimental Section

### 2.1. Materials and Instruments

Tetrachloroauric (HAuCl_4_·4H_2_O, purity ≥ 99.9%), ethyl alcohol, and chlorhydric acid (HCl) were purchased from Sinopharm Chemical Reagent Co., Ltd (Shanghai, China). NaCl, cetyltrimethyl ammonium bromide (CTAB), and trisodium citrate (AR) were from TIANLI Chemical Reagents Ltd (Tianjin, China). Polyacrylic acid terminated by SH (SH-PAAs, Mn = 10^4^ g/mol, Mw/Mn = 1.07) was obtained from Xi’an ruixi Biological Technology Co., Ltd (Xi’an, China). Poly (sodium styrenesulfonate) (PSS, 20 wt.% solution in H_2_O, M.W. = 70,000) was acquired from J&K scientific Ltd (Beijing, China). Milli-Q water was used in all the experiments. All used glassware was treated with aqua regia, rinsed in Milli-Q water, and oven-dried prior to use.

UV-Vis spectra were recorded with a Lambda 35 UV-Vis spectrometer (PerkinElmer, Waltham). Effective surface charges were measured using a ζ potential analyzer (Malvern Instruments Zetasizer, Worcestershire, UK). Field emission scanning electron microscopy (FESEM) images were recorded on a FESEM (Hitachi, SU-8020). Transmission electron microscopy (TEM) images were recorded on a JEM-2100 instrument (JEOL, Tokyo, Japan) with an accelerating voltage of 200 kV (20 μL of the sample was dropped onto a carbon-coated Cu grid and air-dried).

### 2.2. Preparation of a Series of Mixtures for Characterization

Synthesis of citrate-capped AuNPs: The citrate- capped AuNPs (~25 nm in diameter, ~3 × 10^12^ NPs/mL in concentration) were synthesized using a previously reported method [11]. The detailed process about the preparation of the AuNPs and the corresponding size distribution is in the Supplementary Materials (Figure S1).

Preparation of AuNPs solution: Fifty milliliters of prepared AuNPs solution was centrifuged at 4 °C (12,000 rpm, 20 min), diluted to the same volume after removing the supernatant, and denoted as AuNPs.

Preparation of AuNP-PAAs/PAAs solution: AuNPs modified with SH-PAA molecules were prepared by mixing 2 mL of centrifuged AuNPs and 1 mL of SH-PAA molecules aqueous solution (0.004 g/mL) and oscillated for 12 h. The resultant solution was denoted as AuNP-PAAs/PAAs solution (in presence of free PAAs). 

Preparation of AuNP-PAAs/PAAs solution with different pHs: Adjusting the pH of 3 mL of AuNPs-PAAs/PAAs solution by alternately adding 40 μL of 0.5 M HCl and 20 μL of 1 M NaOH 3 times, respectively. Their corresponding UV-Vis spectra and Zeta potentials were recorded after each adjustment.

Preparation of AuNPs-PAAs solution: By centrifuging 3 mL of AuNP-PAAs/PAAs at 4 °C (15,000 rpm, 20 min), the characteristic peak ascribed to PAA appeared in the UV region of the UV-Vis spectrum of the supernatant. By repeating (twice) centrifuging-washing cycles until this peak disappeared, the pH of the solution was determined to be 4. The obtained solution was denoted as AuNPs-PAAs.

Preparation of AuNPs-PAAs solution with different pHs: Introducing 40 μL of 0.5 M of HCl into 3 mL of AuNPs-PAAs solution, the change in the UV-Vis spectrum with time and photographs of the mixture were recorded. When the spectrum and color of the mixture did not change significantly over time, that is, it achieved equilibrium, the pH was measured. Then, 20 μL of 1 M NaOH was added, similar to the operation after adding HCl; the spectrum, color of the solution, and terminal pH at equilibrium were recorded. The acid-base adjustment process was repeated 5 times. 

For comparison, AuNP solutions with different pHs were prepared. Two milliliters of prepared AuNPs solution was centrifuged at 4 °C (12,000 rpm, 20 min) and diluted to the same volume after removing the supernatant. This step was repeated two times and its spectrum was recorded after the addition of 1 mL of MQ water. Then, the pH of the mixture was measured and then adjusted 3 times using 40 μL of 0.5 M HCl and the absorption spectra were monitored.

Preparation of AuNPs-PAAs solution containing PSS molecules: A total of 40 μL of PSS solution (2%) was added three times to 3 mL of AuNPs-PAAs, and the corresponding UV-Vis spectra and Zeta potentials were examined. Then, the pH of the mixture was adjusted to 1.5 and 0.5, in sequence, and their absorption spectra and Zeta potentials were monitored. 

Preparation of AuNPs-PAAs solution containing CTAB: A total of 40 μL of CTAB solution (0.01186 M) was added 17 times to 3 mL of AuNPs-PAAs and the corresponding UV-Vis spectra and Zeta potentials were recorded. Then, the pH of the mixture was measured and then adjusted 7 times using 40 μL of 0.5 M HCl and their absorption spectra and Zeta potentials were monitored. 

Preparation of AuNPs-PAAs ethanol solution: By centrifuging the mixture of AuNPs-PAAs/PAAs at 4 °C (15,000 rpm, 20 min), the obtained residue was diluted using ethanol to 3 mL. Then, the pH of the mixture was adjusted 7 times using 20 μL of NaOH (1 M) and the corresponding spectra were recorded each time. A total of 40 μL of 0.5 M HCl was introduced 9 times in sequence and the same characterizations were repeated.

Preparation of AuNPs-PAAs solution containing NaCl: A total of 40 μL of 0.5 M NaCl was introduced 13 times to 3 mL of AuNPs-PAAs solution, and the corresponding UV-Vis spectra and Zeta potentials were collected. For comparison, the same experiment procedures were carried out for AuNPs.

## 3. Results and Discussion

**Figure 1:**
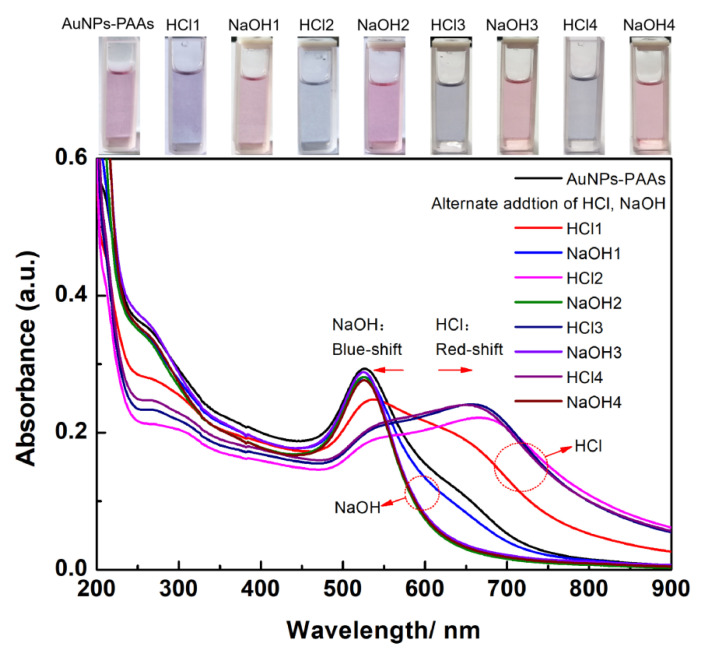
The change in the UV-Vis spectrum of AuNPs-PAAs solution with the alternate adding of acid and base. The numbers after the name of HCl and NaOH represent the sequences of adding HCl and NaOH.

The effect of acidity-basicity on the aggregation behavior of AuNPs-PAAs: Figure 1 shows the change in the UV-Vis spectrum for AuNPs-PAAs solution with acidity and basicity. For AuNPs-PAAs solution, a shoulder peak at 650 nm appears in addition to the characteristic peak of the surface plasmon resonance (SPR) absorption of AuNPs at 530 nm. According to the preparation process of AuNPs-PAAs, the excess SH-PAAs and free citrate in the AuNPs-PAAs solution were removed by centrifugation [12]. As a result, the ionic concentration of the AuNPs-PAAs solution was inevitably decreased. Additionally, this centrifugal separation caused a decrease in pH from about 6 to 4 (being lower than that of pKa of PAA of about 6 [13]). The decrease in the ionic strength and pH caused the Zeta potential of AuNPs-PAAs to decrease [14]. In this case, AuNPs-PAAs could aggregate to some extent, which resulted in the shoulder peak appearance at 650 nm. After addition of HCl for the first time (HCl1), the carboxyl groups of the PAA molecules on the AuNPs further protonated [15] and the electrostatic repulsion between AuNPs-PAAs was reduced. Consequently, the shoulder peak ranging from 600 to 750 nm, representing the aggregated state of AuNPs, was more obvious than that before the addition of HCl, and the SPR absorption peak at 530 nm was related to the dispersed state of AuNPs that shifted towards red and decreased. Interestingly, after introducing NaOH solution (NaOH1), the shoulder peak disappeared, accompanied by the increase in the characteristic SPR peak at 530 nm. This reversed change in the UV-Vis spectrum should be attributed to the deprotonation of PAA; the dispersion of AuNPs-PAAs then increased due to increase in electrostatic repulsion.

In addition to the spectra in the visible region giving information regarding the change in aggregation behavior of AuNPs-PAAs with pH, the UV region provided information about the change in the related species with pH. For the wavelength ranging from 200 to 350 nm, the change in the peak is ascribed to the charge transfer between AuNPs and ligands [16]. It can be seen that the intensity of the peak around 300 nm due to d-d transitions of AuCl_4^−^_ is reversibly dependent on pH [17]. When the pH of the medium is higher than 5, AuCl_4^−^_ is predominant; it transforms to HAuCl_4_ in case of a pH lower than 5 [18]. As a result, the intensity of the shoulder peak located at about 300 nm increased with the addition of NaOH solution, and decreased with the introduction of HCl. 

Additionally, after four cycles of pH adjustment it is clear that this spectral change is completely reversible and that the reversibility enhances with increasing cycles. This may relate to the increased ionic strength, which will be explored later. The above spectral reversible variation is visually reflected by the reversible color changes, the repeated conversion between blue and red, of corresponding samples, shown at the top of Figure 1. Similar phenomena have also been reported [3,19], and the prevailing explanation is the reversible protonation and deprotonation of the carboxylic group of PAAs. To verify that this reversible change of PAAs could cause the reversible change in the electrostatic interaction of AuNPs-PAAs, the Zeta potential of the AuNPs-PAAs solution, before and after the addition of an acid and base, were detected. 

**Figure 2:**
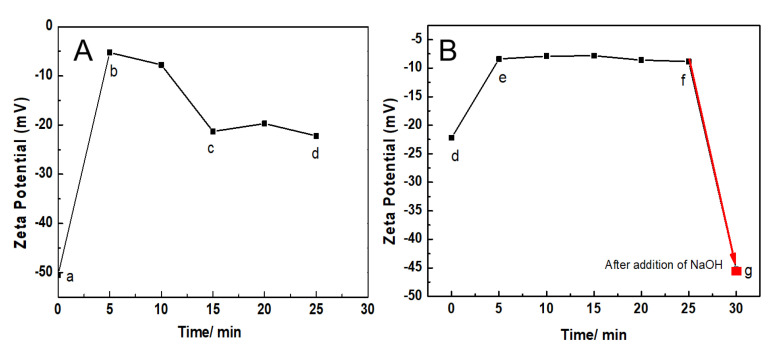
Change in the ζ potential of AuNPs-PAAs with acid-base adjustment. (**A**) AuNPs-PAAs (a) after addition of 40 μL 0.5 M HCl 5 min (b), 15 min (c), 25 min (d). (**B**) Point (d), after addition of 80 μL 0.5 M HCl 5 min (e); point (f), after addition of 20 μL NaOH 5 min (g).

As shown in Figure 2, the Zeta potential of AuNPs-PAAs solution under alternating pH adjustments changed markedly with time, which closely relates to the reversible aggregation of AuNPs-PAAs discussed above. In Figure 2A, the Zeta potential of the AuNPs-PAAs solution climbs from −50 mV (point a) to −5.0 mV (point b) within 5 min after the addition of 40 μL of 0.5 M HCl, and then declines to −20 mV (points c and d). After adding another 40 μL of 0.5 M HCl, as shown in Figure 2B, the Zeta potential of the AuNPs-PAAs solution further increases within 5 min from −20 mV (point d) to −10.0 mV (points e and f). Then, after the addition of 20 μL NaOH, the Zeta potential of the AuNPs-PAAs solution rapidly drops from −20 mV (point f) to −45 mV (point g). The above results obviously indicate that the addition of acid reliably leads to a decrease in the absolute value of Zeta potential, and the addition of alkali results in the opposite result. We found that the presence of PAA layers on AuNPs allows aggregation to have a reversible response to acid and alkali, which can be seen in the Supporting Information (Figure S2). 

The different changing trends of Zeta potential after addition of HCl and NaOH solutions shown in Figure 2A,B are relevant to the physical chemistry properties of AuNPs-PAAs. At the initial stage of adding HCl solution (a and b), the added protons rapidly gathered on the surface of the PAAs layer on the AuNPs-PAAs with higher negative charges (−50 mV). Thus, the absolute value of the Zeta potential rapidly decreased in this stage (from −50 mV (point a) to −15 mV (point b)), and then the protons gradually diffused from the surface to the inside of the PAA palisades to protonate PAAs. Although the protonation of PAA reduces the quantity of charge of AuNPs-PAAs, which reduces the absolute value of the Zeta potential, there are also some factors that could cause an opposite result. In fact, accompanying the decrease in the quantity of charge of AuNPs-PAAs, the positively charged counter ions that adsorbed around the surface of AuNPs-PAAs also decreased, and the charge density of AuNPs-PAAs increased due to the dehydration shrinkage of the protonated segments of PAAs [9,20,21]. This process is relatively slow due to the presence of hydrogen bond networks and the shrinkage in the protonated segments of the PAAs. As a result, the Zeta potential decreased slowly from point b (−5 mV) to point c (−18 mV). 

In particular, if the protonation of PAAs immobilized on AuNPs causes a conformational change, forming a compact structure along the axial direction, it will inevitably lead to a compression of the electric double layer. Consequently, the absolute value of the Zeta potential for AuNPs-PAAs would decrease. However, the experimental results were the opposite. Therefore, we speculated that protonation could not cause a significant conformational change of PAAs anchored to AuNPs.

For the slow decrease in the absolute Zeta potential value from point d (−18 mV) to point e (−10 mV), this was related to the further protonation of AuNPs-PAAs after the first protonation. As previously mentioned, there were hydrogen bond networks and shrinkage in the protonated segments of PAAs after the addition of HCl solution the first time. Therefore, the absolute value of the Zeta potential rapidly achieved stability after dropping to 10 mV due to the second protonation being inhibited by the first protonation. 

For the addition of 20 μL NaOH to the second protonated AuNPs-PAAs solution after 25 min, the Zeta potential of the AuNPs-PAAs rapidly dropped from −10 mV to −45 mV within 5 min (from point f to g). This change was attributed to the fact that NaOH neutralizes, not only the protons adsorbed around the surface of the AuNPs-PAAs, but also the protonated carboxyl groups of PAAs [15]. As a result, the charge of the AuNPs-PAAs increased and the aggregated AuNPs-PAAs solution was re-dispersed due to the increased electrostatic repulsion, and PAAs palisades became loose, which is conducive to OH^−^ diffusion. Thus, the absolute value of the Zeta potential of AuNPs-PAAs increased rapidly.

**Figure 3:**
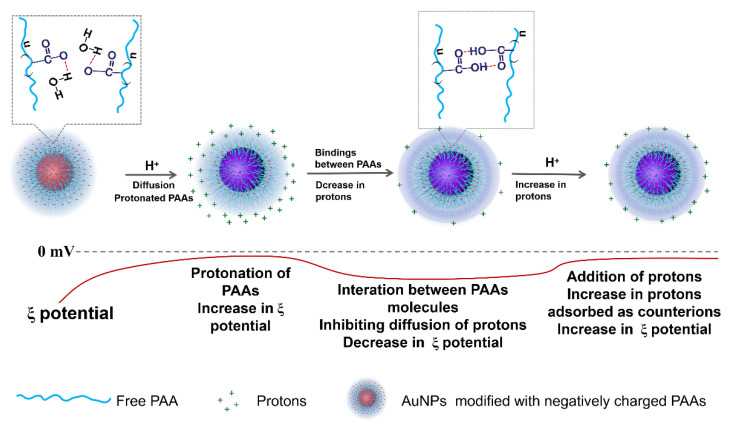
A possible mechanism on the variation of AuNPs-PAAs Zeta potential with addition of acid.

The variational trends of Zeta potential with the addition of acid and alkali well correspond to the explanations for the results of the UV-Vis spectra shown in Figure 1. Namely, the aggregation behaviors of the AuNPs-PAAs are mainly ascribed to the change in electrostatic interactions rather than the conformation of PAAs. Based on the above discussions, we put forward a mechanism of the effect of acid on the Zeta potential of AuNPs-PAAs, as shown in Figure 3. 

**Figure 4:**
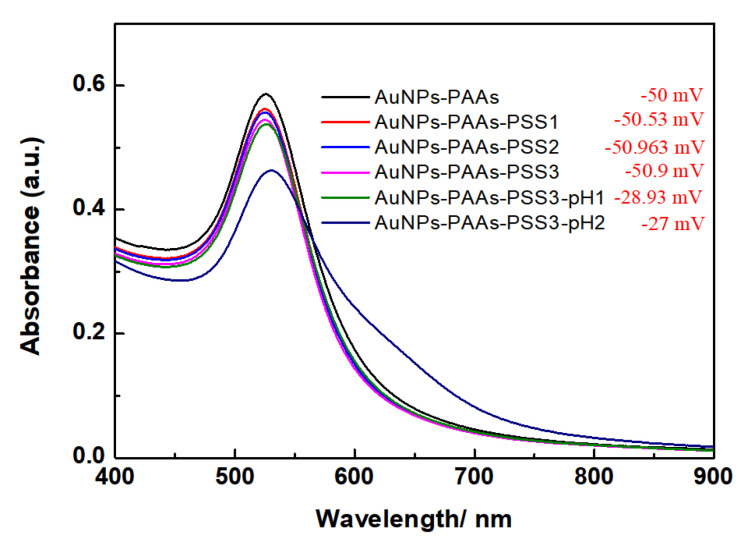
The change in UV-Vis spectra of AuNPs-PAAs with PSS concentration in the presence of HCl (The corresponding Zeta potential values were inserted.). The numbers after the name of PSS represents the sequences of adding PSS solution.

The effect of PSS on the aggregation behavior of AuNPs-PAAs: To verify the change in the charge characteristics of AuNPs-PAAs with acid using the above-proposed mechanism, the interactions between negatively charged PSSs and AuNPs-PAAs in presence of HCl were investigated, and the corresponding results are shown in Figure 4. Clearly, the UV-Vis spectra and the Zeta potential of AuNPs-PAAs hardly change relative to the PSS concentration. This fact agrees with a universal model of electrostatic interaction that there is no interaction between negative charged PAAs and negatively charged PSSs. After the addition of HCl, although the absolute Zeta potential value of AuNPs-PAAs solution containing PSS molecules decreased from 50.9 to 28.93 mV, the corresponding UV-Vis spectrum scarcely changed. After further addition of HCl, the absolute Zeta potential value scarcely changed (from 28.9 mV to 27.0 mV) and a weak shoulder peak ranging from 600 to 700 nm appeared in the corresponding UV-Vis spectrum. Interestingly, for the AuNPs-PAAs solution containing PSS molecules, the absolute Zeta potential value decreased evidently, but the corresponding characteristic UV-Vis absorption peak ascribed to the aggregation of AuNPs-PAAs barely occurred. These findings are completely different from those indicating that the decrease in absolute Zeta potential value leads to the aggregation of AuNPs-PAAs in the absence of PSS molecules. This difference implies that the conformation of PAAs immobilized on the surface of AuNPs in an acidic medium does not change significantly. If the protonated PAAs have a hydrophobically coiled conformation in acidic medium, the interaction between the negative charged PSSs and AuNPs-PAAs is impossible. According to our proposed mechanism, shown in Figure 3, the results shown in Figure 4 could be explained. In an acidic medium, the strong hydrogen bonds among the immobilized PAAs causes them to stand upright on the surface of AuNPs, which could absorb protons to have a certain positive electricity. In this case, the negatively charged PSS molecules could adsorb on the surface of AuNPs-PAAs to inhibit the aggregation of AuNPs-PAAs, even at a lower absolute Zeta potential value. Of course, acidity that is too high leads to a further reduction in absolute Zeta potential value, which is also inevitably causes a slight aggregation of AuNPs-PAAs.

**Figure 5:**
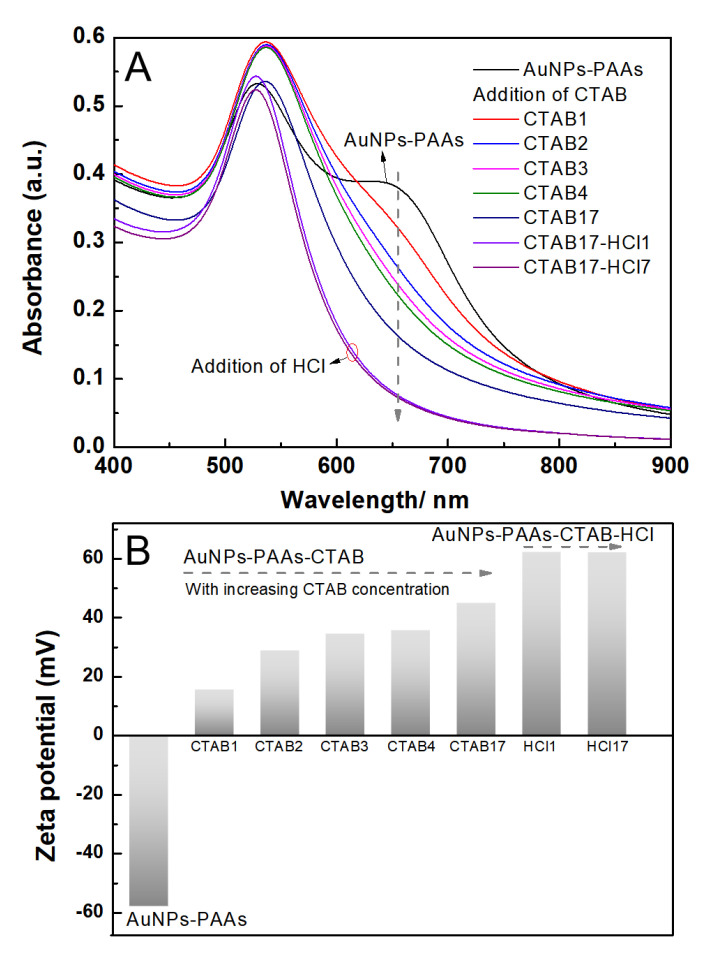
The change in the UV-Vis spectra of AuNPs-PAAs with CTAB concentration and pH (**A**), and the corresponding Zeta potential change (**B**). The numbers after the name of CTAB represent the sequences of adding CTAB solution.

**Figure 6:**
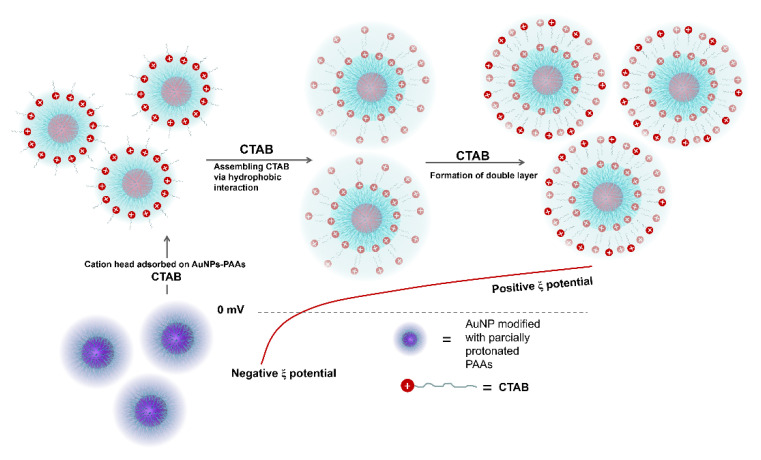
A possible mechanism of effect of CTAB on the Zeta potential of AuNPs-PAAs.

The effect of CTAB on the aggregation behavior of AuNPs-PAAs: To further verify that the immobilized PAAs have no conformational property, the interaction of between the positively charged CTAB molecules and AuNPs-PAAs in presence of HCl was investigated. Figure 5A presents the change in the UV-Vis spectrum of AuNPs-PAAs with CTAB content. As previously described, an obvious shoulder peak appeared around 650 nm in the spectrum for the AuNPs-PAAs solution before adding CTAB. Interestingly, this peak representing the aggregation of AuNPs-PAAs gradually decreased and finally disappeared with increasing CTAB content. After the addition of HCl, the absorbance around 650 nm further decreased. These results implied that PAAs on the surface of AuNPs have no properties of conformational transformation in these cases. If the protonated PAAs have a hydrophobically coiled conformation in acidic medium, the positive charged CTAB could not inhibit the aggregation of AuNPs-PAAs. Based on our proposed mechanism in Figure 3, the results in Figure 5 could be reasonably explained. A study showed that when CTAB molecules with large cationic heads adsorb on a hydrophilic surface, both the hydrophilic environment and steric hindrance could limit its adsorption density. Thus, the conversion from hydrophilic to hydrophobic with the increase in CTAB concentration would not occur [22]. In our opinion, under lower concentrations of CTAB, a few CTAB molecules adsorb sparsely on the surface of AuNPs-PAAs through electrostatic interactions. Then, the tails of the freshly added CTAB molecules insert into sparsely adsorbed alkyl chains through hydrophobic interactions, forming a loose bilayer structure. With the help of hydrophobic interactions, newly added CTAB molecules continue participating in the construction of a CTAB bilayer until forming a compact one. In this process, the charge of AuNPs-PAAs transfers from negative to positive, and the Zeta potential increases with the increase in CTAB content. Consequently, the aggregation of AuNPs-PAAs could be continually restrained. Accordingly, the shoulder peak ranging from 600 to 800 nm constantly decreased. In this case, the negatively charged carboxyl groups of the PAAs were neutralized by introducing HCl, which led to the further increase in the Zeta potential of the AuNPs-PAAs. Accordingly, the shoulder peak ascribed to the aggregation of AuNPs-PAAs disappeared, and the characteristic peak attributed to highly dispersed AuNPs-PAAs appeared. The determined corresponding results of the Zeta potential shown in Figure 5B confirm the above assumption. On the basis of above-discussed points, Figure 6 depicts a proposed effect mechanism for CTAB on the Zeta potential of AuNPs-PAAs.

**Figure 7:**
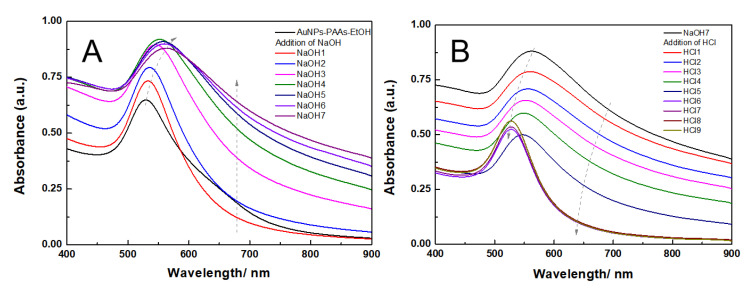
The change in UV-Vis spectra of AuNPs-PAAs in ethanol medium with addition of NaOH (**A**) and HCl (**B**).

The effect of ethanol on the aggregation behavior of AuNPs-PAAs: To reveal the charged properties of AuNPs-PAAs in acidic and alkali medium, the aggregation behavior of AuNPs-PAAs in a lower polar solvent, ethanol, was investigated. Figure 7 shows the change in the UV-Vis spectra of AuNPs-PAAs in ethanol medium with NaOH and HCl concentrations. Figure 7A indicates that the characteristic SPR peak of AuNPs-PAAs in ethanol medium is still located at 520 nm and there is no apparent absorption above 600 nm. This spectral feature is different from that of AuNPs-PAAs aqueous solution, shown in Figure 1. It was reported that ethanol is less capable of accepting protons compared with water [23]. This means that the dissociation ability of PAAs decreases in ethanol, and the absolute value of the Zeta potential decreases. As a result, the aggregation of AuNPs-PAAs should enhance. However, the UV-Vis spectrum in Figure 7A does not show this conjectural phenomenon (black curve), indicating that the special dispersed state of the AuNPs-PAAs in ethanol medium might relate to other reasons. For the AuNPs-PAAs solution used, the pH was around 4. In this case, PAAs were incompletely ionized and ethanol has a solvation effect on them [24,25], accounting for the relatively stable dispersion of AuNPs-PAAs in ethanol [26]. Namely, in this case, the solvation PAAs with extended conformation played a decisive role in the stability of AuNPs-PAAs. 

When NaOH solution was continually introduced, the SPR peak of AuNPs-PAAs was located at around 520 nm and was slightly red-shifted; the absorbance in the long wavelength ranging from 600–900 nm gradually increased. This phenomenon was the opposite of that observed in the corresponding water phase (Figure 1). It has been reported that the electrostatic interaction is inversely proportional to the dielectric constant [27,28]. Therefore, added Na^+^ ions interacting with negatively charged carboxyl groups in PAA chains enhanced its ionic strength, which in turn resulted in the aggregation of AuNPs-PAAs in an ethanol medium.

The change in the UV-Vis spectra of the NaOH-treated AuNPs-PAAs solution with the addition of HCl is shown in Figure 7B. Contrary to adding an alkali solution, with the addition of HCl, the UV-Vis absorbance above 600 nm continuously decreased and the spectral profile finally coincided with that of well-dispersed AuNPs-PAAs. This is because, in this case, hydrogen bonding networks formed between protonated carboxyl groups and ethanol molecules, increasing the solvation effect of ethanol. Thus, the aggregated AuNPs-PAAs gradually redispersed. As a consequence, the spectrum blue-shifted with HCl content in ethanol medium.

**Figure 8:**
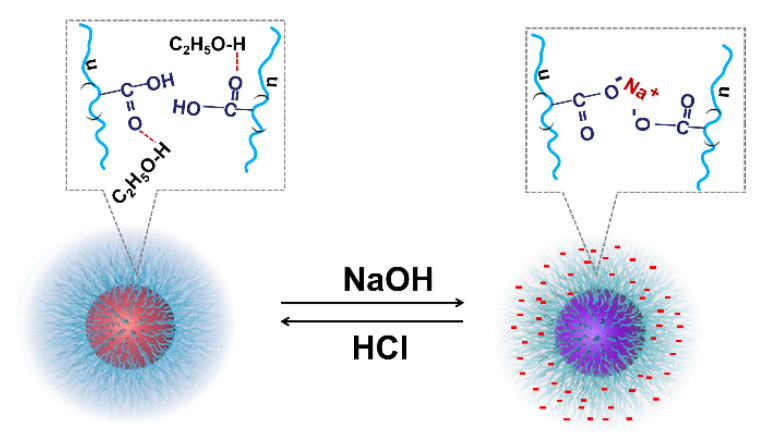
Possible mechanism on the effect of acid and alkali on the aggregation of AuNPs-PAAs in ethanol medium.

It is worth noting that, generally, there is an abrupt change in physical chemistry properties during the conformational transformation of PAAs [29]. However, there is no abrupt change in the UV-Vis spectra of AuNPs-PAAs in ethanol medium in the case of introducing NaOH and HCl. This further verifies that there is no conformational change in this case. According to the above investigation, a possible mechanism on the effects of acid and alkali on the aggregation of AuNPs-PAAs in ethanol medium was proposed and is shown in Figure 8. 

**Figure 9:**
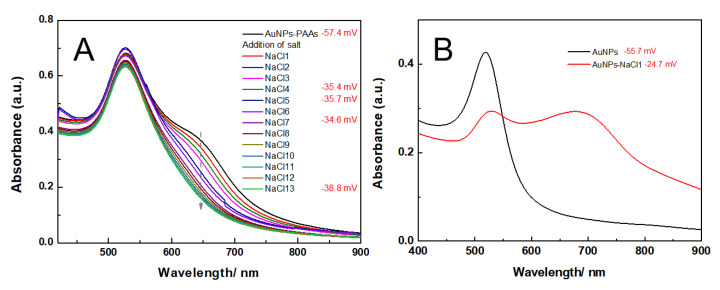
The change in UV-Vis spectra of AuNPs-PAAs (**A**) and AuNPs (**B**) with NaCl concentration and corresponding Zeta potential value.

The effect of salts on the aggregation behavior of AuNPs-PAAs: Considering the effect of salts on PAA conformation change, the UV-Vis spectral changes of AuNPs-PAAs with salts may offer some information on the conformational change of immobilized PAAs, as shown in Figure 9A. As we can see, the intensity of the shoulder peak ranging from 600 nm to 750 nm gradually decreased with increasing NaCl content, implying that the dispersity of the AuNPs-PAAs was enhances. However, the corresponding absolute value of the Zeta potential of the AuNPs-PAAs became lower (insert in Figure 9A). This phenomenon contradicted the general conclusion that reduced absolute potential values strengthen the aggregation of AuNPs. As we all know, the electrostatic screening effect of salt could lead to AuNPs aggregation [30]. The results shown in Figure 9B agree with this conclusion. This abnormal phenomenon could be explained by the following.

After introducing NaCl, Na^+^ ions entering the PAA layer bind to the water molecules bound on the PAAs chains, enhancing intermolecular interactions among PAA chains. Therefore, the rigidity of the PAA chain is enhanced [31,32], which makes it hard to have a conformational change. Most importantly, the concentration of Cl^−1^ ions around the surface of AuNPs-PAAs increases with salt concentration [33,34]. Accordingly, the solvated layer thickness of the AuNPs-PAAs surface increases with increased salt concentration, and the space among the AuNPs-PAAs increased, which is conducive to the dispersion of AuNPs-PAAs. This explanation agrees with the fact that the intensity of the shoulder peak ranging from 600 nm to 750 nm for AuNPs-PAAs solution decreased with increasing salt concentration (Figure 9).

**Figure 10:**
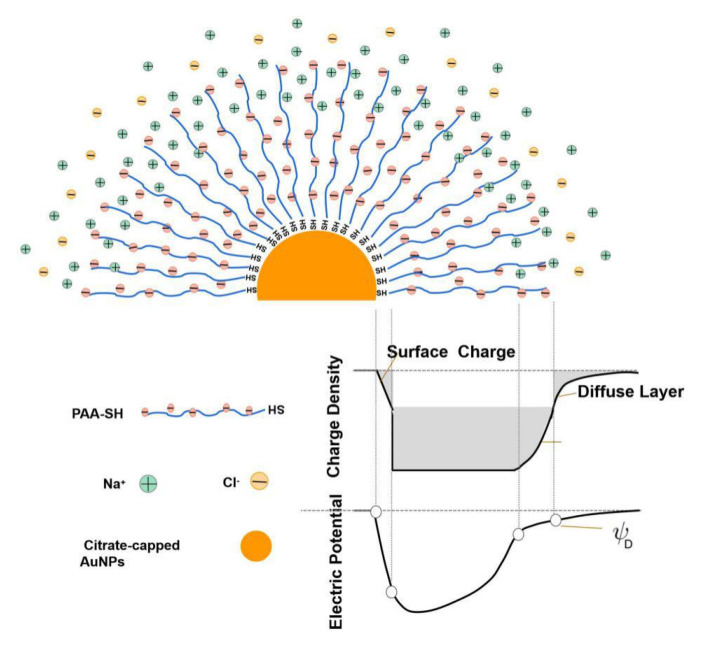
A schematic diagram for the effect of salt on the Zeta potential of AuNPs-PAAs.

As for the detailed changes in the Zeta potential and the UV-Vis spectrum with salt, these findings could be reasonably explained. Although NaCl content continuously increased, the surface potential of the AuNPs-PAAs solution barely changes. Generally, the addition of salt causes more counter ions to adsorb on PAA chains and the net charge of the AuNPs-PAAs decreases [31]. However, here, the Zeta potential generally maintained a constant value of −35 mV when NaCl content further increased. This phenomenon may likely relate to the special property of PAAs immobilized on AuNPs. Unlike free PAAs, for immobilized PAAs, the electrostatic interactions between Na^+^ ions and the negatively charged PAAs is controlled by the interaction between PAAs chains. As previously stated, PAAs are densely loaded on the surface of AuNPs, which limits the diffusion of Na^+^ ions into PAAs palisades on AuNPs. This situation agrees with the Poisson–Boltzmann theory [35]. For the colloid saturatedly loaded with polyelectrolyte, the effective surface charge of the colloid adsorbing more charged species is less than that of bare particles, and the effective surface charge of the polyelectrolyte-immobilized colloid is less liable to be affected by salt concentration. Figure 10 shows a schematic diagram of the effect of salt on the Zeta potential of the AuNPs-PAAs.

The effect of free PAAs on the aggregation behavior of AuNPs-PAAs: In view of free PAAs having the function of pH-dependent conformation change, we expect to explore the interaction between free PAAs and immobilized PAAs on AuNPs to get some useful information regarding the conformation of the immobilized PAAs.

**Figure 11:**
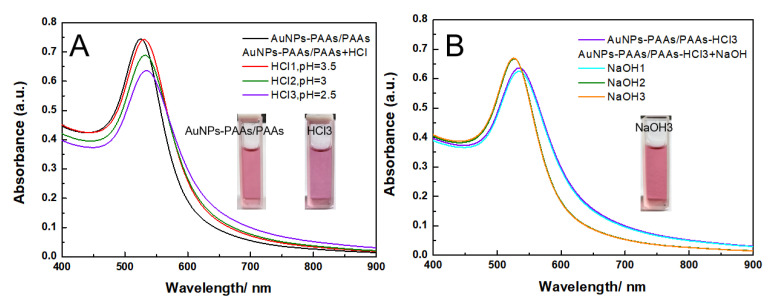
The change in UV-Vis spectra of AuNPs-PAAs with HCl (**A**) and NaOH (**B**) content in the presence of PAAs.

**Figure 12:**
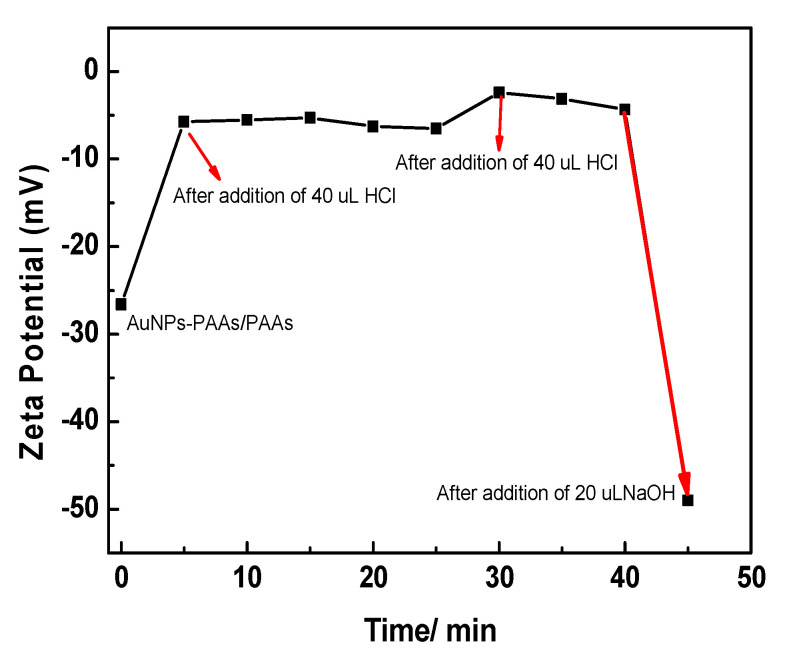
The change in UV-Vis spectra of AuNPs-PAAs in presence of free PAAs with time after adding HCl.

As shown in Figure 11, although there is a reversible change in the UV-Vis spectrum with acid-alkali alternative adjustment for AuNPs-PAAs/PAAs, both the absorbance peak position and the intensity scarcely changes. Even more surprisingly, in acidic medium, although the Zeta potential is around −5 mV (Figure 12), the color of the mixture still remains red (insets in Figure 11) and, the UV-Vis spectral profile does not significantly change. We believe that the special stability of AuNPs-PAAs/PAAs is relevant to the failed conformation change of immobilized PAAs. Because if both the immobilized PAAs and free PAAs had the conformational change in acidic medium, the aggregation of AuNPs-PAAs/PAAs would enhance due to the hydrophobic interaction between the free PAAs and immobilized PAAs.

**Figure 13:**
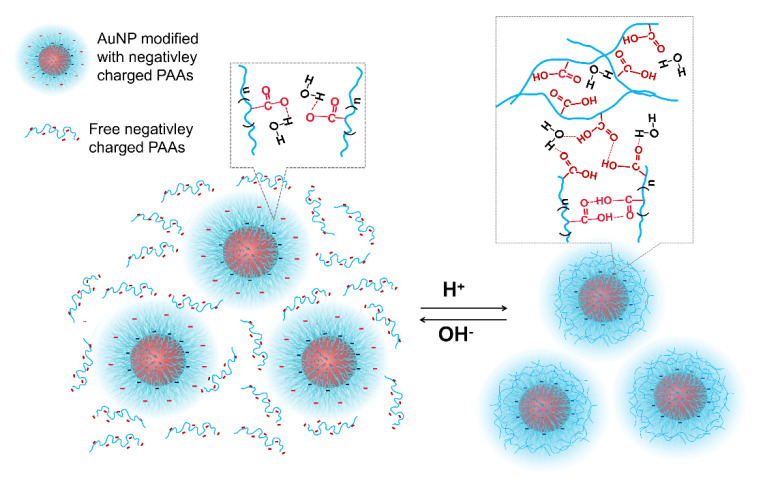
A possible mechanism on the effect of acid-base on the aggregation behavior of AuNPs-PAAs in presence of free PAAs.

As mentioned above, PAAs anchored on AuNPs are more prone to form intermolecular hydrogen bonds in acidic media, making it hard for them to have an obvious conformational change. In this situation, the protonated carboxyl groups on free PAA chains could interact with those on immobilized PAAs on the surface of AuNPs through hydrogen bonds. As a result, the free PAAs are adsorbed on the surface of AuNPs-PAAs, preventing them to not only undergo the conformational change but also AuNPs-PAAs to aggregate. This solvation PAA layer around AuNPs was reportedly ascribed to a certain hydrophilicity of the hydrogen bond network [36]. According to the above discussions, a proposed mechanism on the effects of acid–base on the aggregation behavior of AuNPs-PAAs/PAAs is depicted in Figure 13. The proposed picture for the interaction between AuNPs-PAAs and PAAs in acidic medium agrees with the UV-Vis spectra shown in Figure 11 and the corresponding Zeta potentials shown in Figure 12.

## 4. Conclusions

In summary, to reveal the mechanisms of the reversible color change of polyacrylic acid-modified AuNPs responding to pH, various factors affecting the pH-response of AuNP-PAA solutions, including salt, CTAB, sodium polystyrene sulfonate, ethanol, and free PAA, were investigated. Based on the results, the following conclusions could be drawn. (1) The PAAs terminally immobilized on the surface of AuNPs could respond to pH variations, resulting in a reversible aggregation-to-dispersion change in AuNPs-PAAs. In an aqueous solution, AuNPs-PAAs solution shows an aggregated state (in blue) in acidic medium while a dispersed state (in red) in alkaline medium. This transformation is completely reversible. The aggregation-to-dispersion change of AuNPs-PAAs induced by acidic and alkaline media in an ethanol medium is opposite to that in water. (2) The reversible aggregation of AuNPs-PAAs induced by pH change is attributable to the change in Zeta potential on the surface of AuNPs-PAAs rather than the conformational change of PAAs. Under acidic conditions, the protonation of PAAs reduces the absolute Zeta potential value of AuNPs-PAAs, leading to the aggregation of AuNPs-PAAs. In an alkaline medium, the deprotonation of PAA increases the absolute Zeta potential value of AuNPs-PAAs, resulting in dispersion of AuNPs-PAAs. (3) The factors, salt and CTAB, which could induce aggregation of AuNPs could facilitate the dispersion of AuNPs-PAAs. This difference is attributable to the ionic strength-dependent solvated PAAs layer of the AuNPs. (4) The interaction among immobilized PAAs on AuNPs should not be neglected, because this interaction strongly restrains their conformational change. (5) The existence of free PAAs in AuNPs-PAAs solution significantly inhibits pH response compared with that of AuNPs-PAAs. The formation of the free PAA adsorbed layer on AuNPs-PAAs inhibits, not only their own conformational change, but also the aggregation of AuNPs-PAAs. (6) The aggregation of AuNPs-PAAs depends, not only on the Zeta potential, but also on the thickness of the solvation layer around the AuNPs. The protocols and the findings involved in this investigation give some new insights to understand the pH-response of AuNPs-PAAs and the design of AuNPs-PAAs-based functional sensors.

## Supplementary Materials

The following are available online at https://www.mdpi.com/article/10.3390/ma14133679/s1, Figure S1: TEM images for AuNPs (A) and corresponding size distribution for AuNPs (B), Figure S2: Photographs of the dried aggregates for AuNPs-PAAs (A) and AuNPs (B) before (left) and after adding NaOH solution (right), Figure S3: SEM images for the dense aggregates formed by AuNPs-PAAs (A) and AuNPs (B), and TEM images for the corresponding sample A (C) and B (D) after NaOH solution treatment.
