# Supplementary material for: Study on the Mechanism of the Reversible Color Change of Polyacrylic Acid Modified Gold Nanoparticles Responding to pH
Source: Materials (Basel). 2021 Jul 1;14(13):3679. doi: 10.3390/ma14133679 (PMC8269886; doi:10.3390/ma14133679)
Supplement: Supplementary file 1 [file materials-14-03679-s001.zip › materials-1232435-supplementary.pdf]

Supplementary Materials

Study on the Mechanism of the Reversible Color Change of Polyacrylic Acid Modified Gold Nanoparticles Responding to pH

Runmei Li, Caixia Zhang, Chen Wang, Yongjuan Cheng and Daodao Hu*

Engineering Research Center of Historical and Cultural Heritage Protection, Ministry of Education, School of Materials Science and Engineering, Shaanxi Normal University, Xi'an 710062, China; lrm@snnu.edu.cn (R.L.); zxc@snnu.edu.cn (C.Z.); ccw1128@snnu.edu.cn (C.W.); CYJ04240045@163.com (Y.C.)

* Correspondence: daodaohu@snnu.edu.cn

Figure S1

Synthesis of citrate-capped AuNPs. A total of 25 nm of citrate capped AuNPs were synthesized using a previously reported method [1]. The detailed process is as follows. First, AuNP seeds were prepared. A total of 150 mL Milli-Q water containing 2.2 mM sodium citrate in a 250 mL three-necked round-bottomed flask was heated for 15 min under vigorous stirring. After boiling commenced, 1 mL of HAuCl4 (25 mM) was injected. The color of the solution changed from yellow to bluish gray and then to soft pink in 10 min. The resulting particles (～10 nm, ～3×1012 NPs/mL) were coated with negatively charged citrate ions and hence were well suspended in H2O. Second, AuNPs 25 nm in diameter were prepared through the seeded growth method. The AuNP seed solution was cooled until the temperature of the solution reached 90 °C. After extracting 2 mL of AuNPs seeds solution, 1 mL of a HAuCl4 solution (25 mM) was injected for 30 min reaction. After repeating this process, the solution was cooled to room temperature, generating AuNPs with a size of 25 nm (～3×1012 NPs/mL in concentration). The size distribution of the prepared citrate-capped AuNPs is shown in Figure S1. The particle molar concentration could be calculated according to the following equation [2].

$$C_n = \frac{A_{max} \ln 10 \times 10^3}{\prod r^2 \, Q_{ext} d_0 N_A}$$

where r is the particle radius, Q_{ext} is the extinction efficiency (Q_{ext} = 394,357× r (nm)), d_0 is the path length of the spectrometer, and N_A is Avogadro constant.

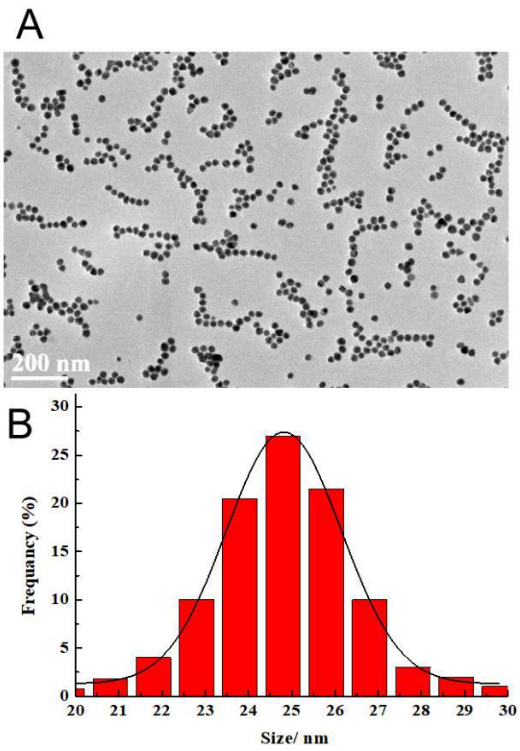

Figure S1. TEM images for AuNPs (**A**) and corresponding size distribution for AuNPs (**B**).

Figure S2

The golden aggregations of AuNPs and AuNPs-PAAs. One interesting thing is that black aggregates were formed on the bottom of the centrifuge tubes after centrifugation of both AuNPs-PAAs/PAAs and AuNP solution, and these aggregates became golden after drying in air (Figure S2).

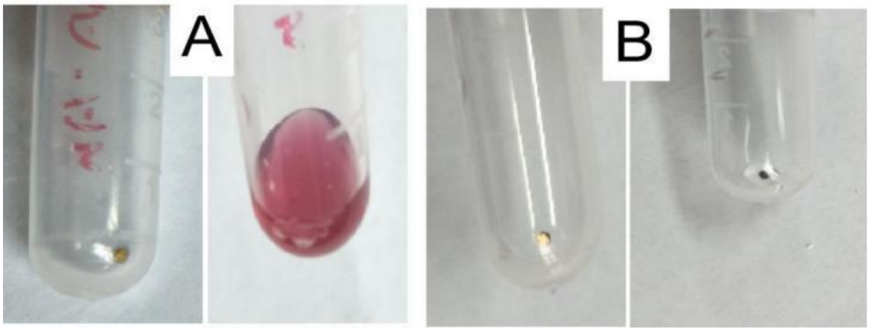

Figure S2. Photographs of the dried aggregates for AuNPs-PAAs (**A**) and AuNPs (**B**) before (left) and after adding NaOH solution (right).

Generally, the color of AuNP aggregates depends on the distance between AuNPs. For AuNPs-PAAs, both centrifugation and the reduced charges of AuNPs-PAAs caused by the protonation of immobilized PAAs were enhanced (Figure S1). TEM images for AuNPs (A) and corresponding size distribution for AuNPs (B) aggregation. As a consequence, the distance between the AuNPs-PAAs decreases and their aggregates exhibit a black color. When water molecules in the aggregates volatilize, AuNPs-PAAs get closer, forming a tightly packed structure. As we can see from the SEM image in Figure S2A the AuNPs-PAAs particles were closely packed. Although AuNPs-PAAs particles

are in an aggregated state, there is a clear interface among the particles. However, for AuNPs, there is a coalescence of particles (Figure S2B), indicating that the interface between AuNPs particles disappears due to the interfacial reaction between bare AuNPs [3]. The above results suggest that the immobilized PAAs on AuNPs could inhibit the fusing between AuNPs. It is this effect that allows the re-dispersion of AuNPs-PAAs aggregates with a golden color in NaOH solution (Figure S2A). Instead, AuNPs aggregates could not re-disperse in NaOH solution (Figure S2B).

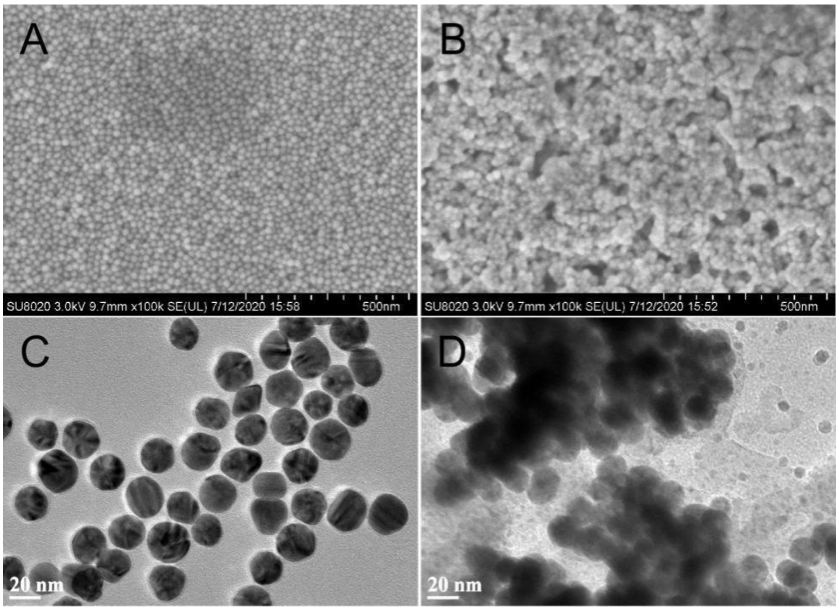

Figure S3. SEM images for the dense aggregates formed by AuNPs-PAAs (**A**) and AuNPs (**B**), and TEM images for the corresponding sample A (**C**) and B (**D**) after NaOH solution treatment.

Interestingly, the dried aggregates of AuNPs-PAAs had a golden color. For bulk gold, there is an extremely high electron density, and the electronic states are densely spaced, forming quasi-continuous bands, which determines its unique golden color [4]. For AuNP aggregates with a coalesced structure, their electrons behave like the free electrons of bulk gold. This leads to the glowing property of the aggregated AuNPs. However, for AuNPs-PAAs, although there are obvious Figure.S2-2 SEM images for the dense aggregates formed by AuNPs-PAAs (A) and AuNPs (B), and TEM images for the corresponding sample A (C) and B (D) after NaOH solution treatment. interfaces between AuNPs-PAAs, the aggregates still present the golden color. This result implies that the film formed by the aggregates of AuNPs-PAAs has electrical conductivity. The study pointed out that this unusual phenomenon is closely related to electron tunneling induced by strong LSPR coupling among AuNPs-PAAs [5].

References

1. Bastus, N. G.; Comenge, J.; Puntes, V., Kinetically controlled seeded growth synthesis of citrate-stabilized gold nanoparticles of up to 200 nm: size focusing versus Ostwald ripening. Langmuir 2011, 27 (17), 11098-11105.
2. Tang J. Q; Gao K. P.; Ou Q. H.; Fu X. W.; Man S. Q.; Guo J.; Liu Y. K., Spectrochimica Acta Part A: Molecular and Biomolecular Spectroscopy. 2018, 191, 513–520.
3 Ghosh, S. K.; Pal, T., Interparticle Coupling Effect on the Surface Plasmon Resonance of Gold Nanoparticles: From Theory to Applications. Chemical Reviews 2007, 107 (11), 4797-4862.

4. Li, C.; Cahen, D.; Wang, P.; Li, H.; Zhang, J.; Jin, Y., Plasmonics Yields Efficient Electron Transport via Assembly of Shell-Insulated Au Nanoparticles. iScience 2018, 8, 213-221.
5. Ebrahimpour, Z.; Mansour, N., Plasmonic Near-Field Effect on Visible and Near-Infrared Emissions from Self-Assembled Gold Nanoparticle Films. Plasmonics 2017, 13 (4), 1335-1342.
